# Monitoring PRRSV-1 in suckling piglets in an endemic herd using reverse transcriptase quantitative real time polymerase chain reaction: comparison of the rate of detection in serum and oral fluid samples and evaluation of pooling

**DOI:** 10.1186/s40813-019-0115-z

**Published:** 2019-02-19

**Authors:** Arnaud Lebret, Gwenaël Boulbria, Pauline Berton, Pierre-Yves Moalic, Jean Le Guennec, Franck Bouchet, Vincent Auvigne, Valérie Normand

**Affiliations:** 1 Porc. Spective Swine Vet Pratice, Chene Vert Conseil veterinary group, ZA du Gohélève, 56920 Noyal-Pontivy, France; 2 Labofarm Finalab Veterinary Laboratory Group, 4 rue Théodore Botrel, 22600 Loudéac, France; 3 Ekipaj, 22 rue d’Assas, 49000 Angers, France

**Keywords:** Pig, Diagnostic, Suckling piglets, Oral fluid, Pooling, PRRS, Serum, PCR

## Abstract

**Background:**

Defining shedding and exposure status for PRRSV is essential in herd stabilisation protocols and weaning-age pigs is a key subpopulation. Oral fluid (OF) sampling is a welfare-friendly and cost saving promising alternative to blood sampling. The first objective of our study was to compare the rate of detection of PRRSV-1 in individual serum sample, individual OF sample, litter-based OF sample, collected the day before weaning. The second objective was to evaluate the interest of pooling samples.

**Results:**

The study was performed on a 210-sows, PRRSV-1 exposed, with confirmed shedding, non-vaccinated against PRRSV, herd. 80 litters were sampled and 26 were viropositive and therefore included. The rate of detection of PRRSV-1 with RT-qrtPCR in blood samples, iOF and cOF was 67, 23 and 77%, respectively. The Ct values from RT-qrtPCR on collective OF were statistically lower if the serum of the piglet of the litter was positive. The lower the Cycle threshold (Ct) value of RT-qrtPCR on collective OF, the higher the probability that the serum sampled in the same litter was positive. Ability to detect PRRSV RNA after pooling was 67% for sera and 58% for cOF.

**Conclusions:**

The rate of detection of PRRSV-1 was about the same in cOF and blood samples. Virus sequencing, if required, should be performed on individual serum samples. The smaller the Ct of a cOF sample from a litter, the greater the likelihood that the serum sample from a piglet of that litter is positive.

A cost-effective and representative sampling protocol to monitor sow herds stabilisation of a sow batch could be: to collect both cOF and one serum sample per litter; to perform firstly RT-qrtPCR on pooled cOF; in case of negative results to consider the batch negative; in case of positive results in a unvaccinated herd or a killed vaccine vaccinated one to consider the batch positive; in case of positive result in a herd vaccinated with a modified live vaccine serum samples of litters with positive cOF should be tested for sequencing (selecting the litters with the lowest Ct for cOF).

## Background

Porcine Reproductive and Respiratory Syndrome (PRRS) is caused by a virus of the *Arteriviridae* family known as PRRS virus (PRRSV) and has become enzootic in most pig production areas [1]. It has a dramatic impact on the health and welfare of pigs, making it the number one enemy of the swine industry worldwide. In the USA, production losses due to the disease were estimated to reach US$ 560 million per year [2]. More recently, the median annual loss per sow per year was estimated to range from €101 up to €650, depending on the PRRS disease scenario on the farm [3].

PRRSV strains can be genetically differentiated into PRRSV-1 (mainly predominant in European countries) and PRRSV-2 (mainly predominant in North America and Asia) [4, 5]. In France, only closely-related PRRSV-1 strains have been isolated until now [6, 7].

Defining the herd status for PRRSV is essential for veterinary practitioners when designing and monitoring herd stabilisation protocols. Herd classification is based on both the shedding and exposure status of the herd. Assessing stability in breeding herds is important [8]. Polymerase Chain Reaction (PCR) will help determine shedding of the herd while antibody-based tests help to determine herd exposure. In sow herds vaccinated with a modified live vaccine (MLV), detectable viraemia in pigs can be associated with either vaccine strains or wild-type virulent PRRSV strains. In these cases, sequencing of PRRSV detected in the herd will allow identifying the strain by sequencing of genes encoding open reading frames (ORF) five and seven (ORF5, ORF7).

A relevant subpopulation in pig herds are weaning-age pigs [8]. Prior to weaning, piglets can be infected by vertical transmission from viraemic sows, either during gestation (around 90 days of gestation) or by nose-to-nose contact [9]. Studying viraemia in piglets prior to weaning is therefore a good approach to monitor PRRSV shedding in sow herds. In PRRSV-positive herds, the lack of detectable viraemia in 30 weaning-age pigs from 4 different farrowing batches in a 3-month period is required to classify the herd status as positive stable [8].

Pathogen surveillance in commercial pig herds is limited by labour costs and the time needed for sampling. The goal is to optimize the pathogen detection capacity with minimal sampling and testing costs. The collection of oral fluid (OF), defined as ‘the fluid obtained by the insertion of absorptive collectors in the mouth’ [10], seems to be a promising option. OF is a mixture of salivary gland and oral mucosal secretions [11] with a composition similar to that of serum [12]. Although this type of sampling was introduced to swine practice relatively recently, it has been widely studied, as the potential for OF based-diagnostics is substantial [13]. OF sampling provides a welfare-friendly method for monitoring swine pathogens [14]. Samples are easy to obtain with non-invasive procedures and can be collected by a single person. OF samples may be collected at both individual or group (pen) level. The feasibility of individual OF sampling has been studied for boars [15], sows [16, 17] and growing pigs [18]. Pen-based OF sampling uses the natural behaviour of pigs [19] and their need to explore their environment [20]. The feasibility of pen-based OF sampling has been demonstrated for growing pigs [21, 22] and sows [23].

Serum collected from individual pigs was long considered as the best diagnostic sample for the monitoring and surveillance of PRRS [4]. As blood sampling is intrusive and stressful, alternative sampling methods have been proposed. Recent research has shown that PRRSV nucleic acids and antibodies can be detected in porcine OF [4, 13, 23] and processed fluids [24]. However, to our knowledge, only one study has evaluated the detection of PRRSV by PCR in weaning-age pigs using OF samples [25]. That study, performed in US sow herds, evaluated the transmission of North American PRRSV strains. It was conducted on PRRSV-vaccinated commercial swine farms and compared PRRSV detection in OF collected at litter level 1 day prior to weaning with PRRSV detection in serum of the sows 2 days post-weaning.

The first objective of our study was to compare the rate of detection of PRRSV in pigs using three different types of samples (individual serum sample, individual OF sample, litter-based OF sample), collected the day before weaning in an unvaccinated PRRSV-1 positive unstable herd. The second objective was to evaluate the interest of pooling samples in batches of five.

## Material and methods

### Study design

#### The study was observational and cross-sectional

The study was performed on a 210-sow farrow-to-finish herd in Brittany, France. Sows were allocated into 7 batches of 30 sows each. Piglets were weaned at around 28 days of age. The herd was PRRSV positive. In suckling piglets, circulation of a PRRSV-1 strain was confirmed by RT-qrtPCR and sequencing in December 2016. No vaccination against PRRSV was performed in this herd.

The study units were the batch and the litter. Four non-consecutive batches were included. In each batch, all litters were sampled (except for batch 4 where 20 litters were selected randomly).

### Sampling and pooling

Samples were collected simultaneously on the day prior to weaning. For each litter, we collected the following samples: blood sample and individual OF (iOF) sample from the weakest piglet in the litter; collective OF (cOF) sample from the litter. Blood samples were collected from the cranial vena cava in plain test tubes and centrifuged at the laboratory to separate serum. In the same piglet, individual OF was collected by allowing the piglet to chew one dry cotton device. The cOF sample was collected using an untreated 100% cotton rope that was presented to the piglets of the litter without prior training. One end of a 50 cm rope (0.8 cm diameter) was knotted and attached with pliers to the middle of the farrowing crate wall. The other end (unknotted) arrived at shoulder level of the smallest piglet. After 30 min, the lower (wet) portion of the rope was inserted into a disposable plastic bag. The rope was manually wrung out inside the plastic bag to release the oral fluid, after which a corner of the bag was cut and the sample was transferred into a 10 ml tube, and kept in cool storage until submission to the laboratory. To avoid cross-contamination between samples, gloves were changed between each cOF sample manipulation.

Samples were collected between December 2016 and August 2017. A total of 4 batches and 110 litters were sampled (30, 30, 30 and 20 litters per batch).

For the evaluation of pooling, in each batch, samples constituting the pool were randomised at the laboratory. A pool of 5 serum samples represented 5 pigs and a pool of 5 cOF at least 50 ones.

### Laboratory analysis

Reverse transcription quantitative real-time PCR (RT-qrtPCR) (Labofarm, Finalab Veterinary Laboratories Group, Loudéac, France) was performed on OF and blood samples, both individual and pooled samples. In case of a positive result at batch level, ORF 5 and ORF 7 sequencing was performed for each sample type and for individual tests and pools. We decided to sequence the sample with the lowest cycle threshold (Ct).

#### Detection of PRRSV by reverse transcription real-time PCR

Before ribonucleic acid (RNA) extraction, cOF samples were clarified by centrifugation at 1000 g for 10 minutes. The supernatants were used for the analysis. The iOF samples were not pre-treated. Samples (OF or sera) were pooled by mixing and vortexing five individual 100 μL samples. RT-qrtPCR was performed on both individual and pooled samples. The total RNA was extracted from OF and sera using QIAamp RNA miniKit (Qiagen, Venlo, The Netherlands) following the manufacturer’s protocol. 140 μL of sample were added to 560 μl of lysis buffer and incubated for 10 minutes at room temperature. At the end of the protocol, total RNA was eluted in 50 μl of AE buffer and kept at − 20 °C until use. PRRSV was detected using ORF7 in-house specific primers and probes. Amplification was performed using an AgPath-ID One-Step RT-rt-PCR kit (Ambion, Thermofisher Scientific, Gent, Belgium) and ARIA MX Real Time PCR system (Agilent Technologies, Les Ulis, France). For each assay, positive and negative controls were tested with field samples. Samples with a Ct lower than 40 and curve showing specific exponential shape were considered as positive. Theoretically we assumed that the lower was the Ct, the higher was the viral nucleic acid quantity in the sample.

#### ORF 7 and ORF 5 sequencing

ORF7 and ORF5 were amplified using specific primers [26, 27]. Fragments of 672-base-pairs (bp) and 734 bp were amplified for ORF7 and ORF5, respectively. PCR was performed using the Qiagen multiplex PCR kit (Qiagen, Venlo, The Netherlands) in a total volume of 25 μL containing 20 g of DNA template, 12.5 μL of Qiagen PCR Master Mix, 2.5 μL of Qiagen Q-solution and 2.5 μL of primer mix at 10 μM each. PCR tests were carried out using a SimpliAmp thermal cycler under the following conditions: initial denaturation at 95 °C for 15 min, followed by 35 cycles of denaturation at 95 °C for 30 s, annealing at 52 °C for 30 s, and extension at 72 °C for 90 s, with a final extension step at 60 °C during 30 min. The amplification success was assessed by electrophoresis on LabChip GX Analyser (Caliper Life Sciences, Hopkinton, USA). Successful PCR products were purified using AMPure beads (Beckman Coulter, Brea, USA) before sequencing using the ABI Big-Dye Terminator v3.1 Cycle Sequencing Kit following the manufacturer’s protocol (Thermofisher Scientific, Gent, Belgium) and finally sequenced on an ABI 3130xl DNA sequencer (Applied Biosystems, Thermofisher Scientific, Gent, Belgium).

### Statistical analysis

To assess the ability of the three index tests (one index for each kind of sample) to detect PRRSV-1 RNA, the result of each test was compared to a reference standard. We defined the reference standard at study unit level (batch or litter) as the cumulative result of the three samples tested. Thus, a litter or a batch was considered positive if at least one of the three samples was positive. This design implicitly considered that the specificity of the RT-qrtPCR was 100%, irrespective of the type of sample.

To assess the efficiency of pooling, the result of each pool of five samples was compared to the individual analysis of these five samples, the individual analysis being the reference. The individual analysis of the five samples was considered positive if at least one of them was positive.

Statistical analyses were conducted using R programming language 3.4.1. The exact binomial test 95% was used to compute the confidence intervals (CI) of rate of detection. The Wilcoxon rank sum test with continuity was used to compare Ct between groups.

## Results

### Ability to detect PRRSV in three types of samples

Three out of the four tested batches had at least one positive sample. RT-qrtPCR on both serum and cOF samples was able to detect at least one positive litter in each batch. RT-qrtPCR on iOF was unable to detect PRRSV in one batch out of 3 (Table 1).

**Table 1:** Results of the three index tests at batch and litter level

		Number of PRRSV RNA-positive samples			
Batch	Number of litters	serum	Collective Oral Fluid	Individual Oral Fluid	Standard
1	30	0	0	0	0
2	30	4	6	4	8
3	30	7	7	2	11
4	20	5	7	0	7

The reference standard is the cumulative result of the three samples tested

26 litters were tested positive out of 80 sampled in the three positive batches. The rate of detection in a given index test was defined as its capacity to detect those 26 positive litters. In blood samples, iOF and cOF, it was calculated at 67, 23 and 77%, respectively. By taking both blood samples and cOF into account, this allowed to detect 96% of the positive litters (Table 2). Because of this poor rate of detection of the virus in iOF samples, results with this type of sampling are not presented in this paper.

**Table 2:** Rate of detection of PRRSV RNA in the three index tests at litter level

	Number of PRRSV RNA-positive samples			
	Index test	Standard	Rate of detection	95% Confidence Interval
Serum	16	26	62%	41–80
Individual Oral Fluid	6	26	23%	9–44
Collective Oral Fluid	20	26	77%	56–91
Serum + collective Oral Fluid	25	26	96%	80–100

The reference standard is the cumulative result of the three samples tested

### Comparative viral load in samples

**Fig. 1:**
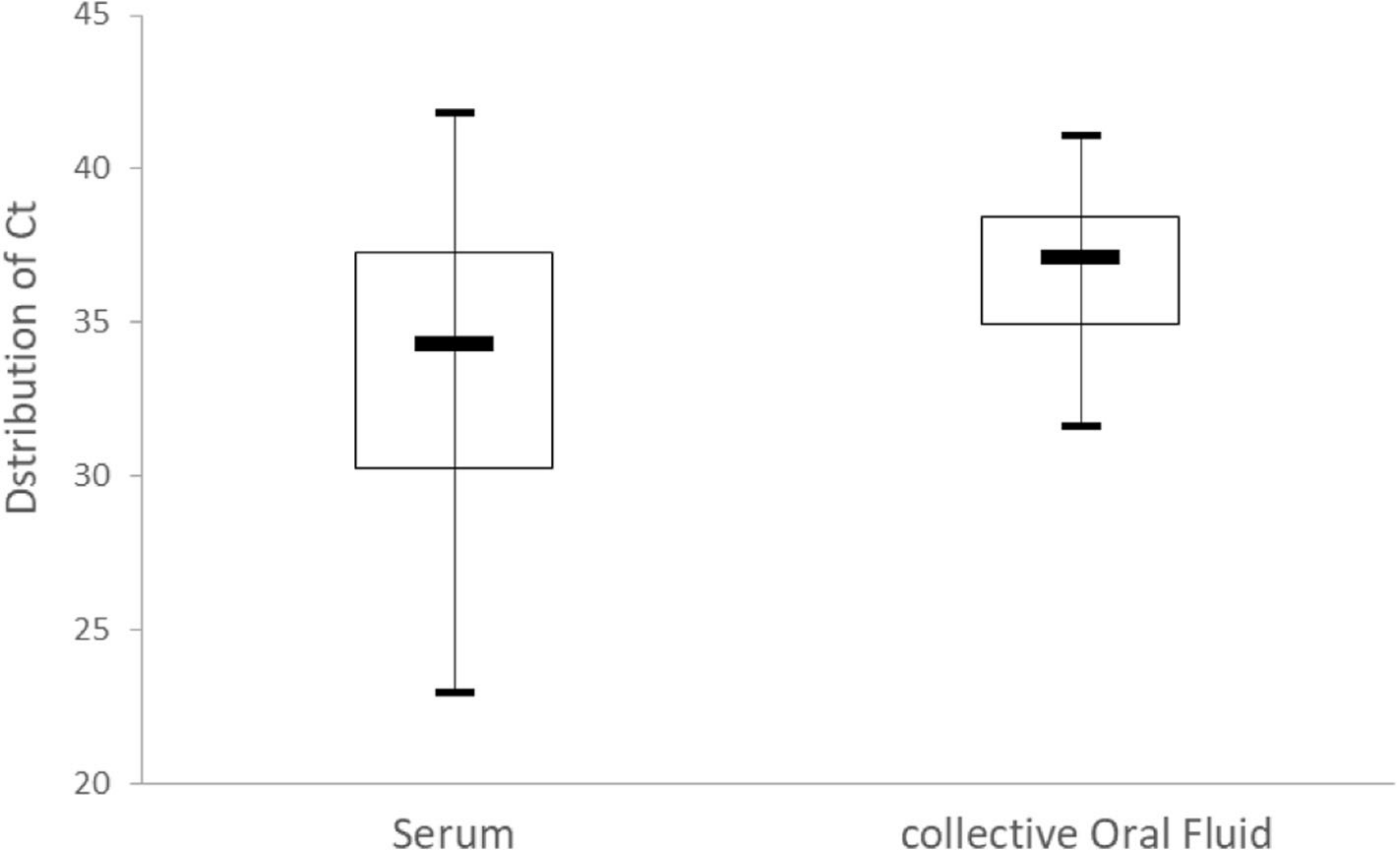
Distribution of Cycle threshold (Ct) values of non-pooled analysis for detection of PRRSV RNA using RT-qrtPCR. Boxplots show median, quartiles, minimum and maximum values

Ct values were available for all positive results (16 sera and 20 cOF). The lowest Ct value was 22.9 for RT-qrtPCR on serum and 31.6 on cOF (Fig. 1). There was a clear statistical tendency of lower Ct values from RT-qrtPCR on serum than on cOF (Wilcoxon test, *p* = 0.06).

**Fig. 2:**
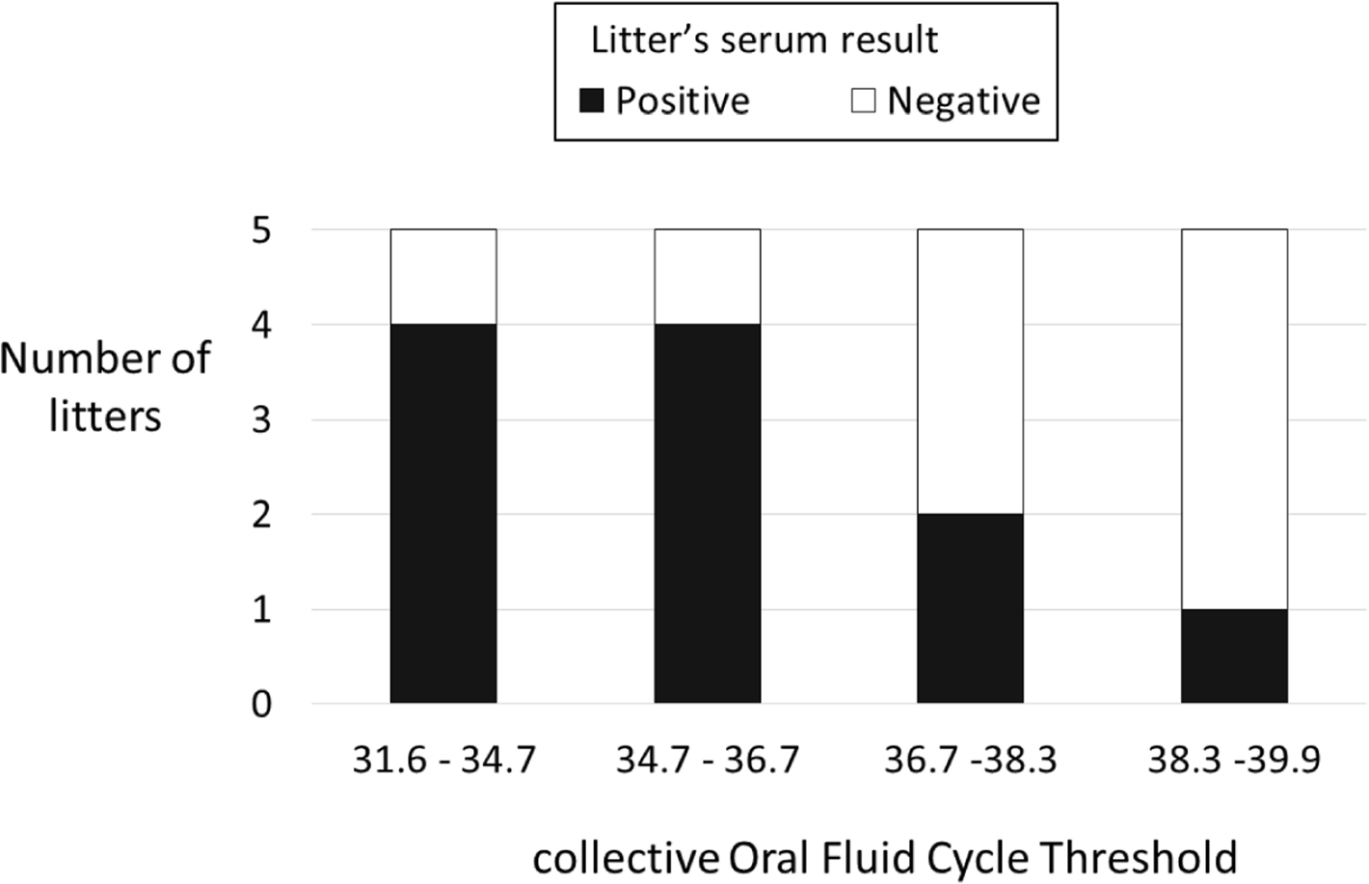
Qualitative result of the serum according to Cycle thresholds (Ct) of RT-qrtPCR on collective Oral Fluid of litter

The Ct values from RT-qrtPCR on cOF were statistically lower if the serum of the piglet of the litter was positive (Wilcoxon test, *p* = 0.02, Table 3). With a lower Ct value of RT-qrtPCR on cOF, the probability that the serum sampled in the same litter was positive was consistently higher: for litters with a Ct value of RT-qrtPCR on cOF over 36.7 only 20% of the piglet serum was positive, whereas for litters with a Ct value below 36.7 (included), 80% of the piglet serum was positive (Fig. 2). This relation is not symmetric: the Ct values from RT-qrtPCR on serum were not statistically different given the qualitative result of the RT-qrtPCR on cOF (Wilcoxon test, *p* = 0.58, Table 4).

**Table 3:** Cycle thresholds (Ct) of collective Oral Fluid (cOF) according to the qualitative result of the serum of the litter for detection of PRRSV RNA using RT-qrtPCR (non-pooled analysis, *p*-value = 0.02)

		Ct of RT-qrtPCR on cOF					
		Minimum	1st Quartile	Median	Mean	3rd Quartile	Maximum
PRRSV RNA detection on serum	Negative	33.3	37.1	38.2	37.7	38.8	39.9
	Positive	31.6	34.4	35.6	35.5	36.6	38.5

**Table 4:** Cycle thresholds (Ct) of the serum of the litter according to the qualitative result of the collective oral fluid (cOF) (non-pooled PCR, *p*-value = 0.58)

		Ct of RT-qrtPCR on serum					
		Minimum	1st Quartile	Median	Mean	3rd Quartile	Maximum
PRRSV RNA detection on cOF	Negative	26.8	34.4	34.6	34.6	37	40.3
	Positive	22.9	29.9	33.6	33.1	36.8	41.8

### Ability to detect PRRSV in pools of five samples

A total of 22 serum pools and 22 cOF pools were constituted. Ten of the serum pools and 10 of the cOF pools were constituted of negative samples only and these 20 pools were tested negative. There was therefore no lack of specificity by pooling. 12 of the sera pools and 12 of the cOF pools were made up of samples with at least one of which was positive. Ability to detect PRRSV RNA after pooling was 67% for sera and 58% for cOF (Table 5). The Ct values of RT-qrtPCR on positive pooled sera ranged from 24.8 to 36.3. The Ct values of RT-qrtPCR on positive pooled cOF ranged from 35.1 to 39.4.

**Table 5:** Ability to detect PRRSV RNA after pooling

	Number of pools			
	Positive	With at least one positive sample	PRRSV RNA detection rate	95% Confidence Interval
Serum	8	12	67%	35–90
cOF	7	12	58%	28–85

For each sampling method, the reference was the presence of at least one positive sample in the pool

The probability for a pool to be tested positive increased with the number of positive samples in the pool. All the pools (*n* = 3) of serum samples and five of the six pools of cOF samples with at least two positive individual samples were positive. However, only five out of nine pools of serum and two out of six pools of cOF samples with only one positive individual sample were positive. For the three positive batches, at least one of the pool analyses was positive, either for serum pools or cOF pools (Table 6).

**Table 6:** Ability to detect PRRSV RNA at the batch level using RT-qrtPCR on sera and collective Oral Fluid (cOF) pooled by five

			Number of positive pools	
Batch	Status	Number of Pools	serum	cOF
1	Negative	6	0	0
2	Positive	6	2	3
3	Positive	6	4	1
4	Positive	4	2	3

### Sequencing

Sequencing from individual sera was successful for all three batches for ORF7 and one batch for ORF5. None of the cOF samples (whether from individual or pooled samples) or of the pooled serum samples could be successfully sequenced.

## Discussion

Pathogen surveillance on commercial swine farms provides information for veterinary practitioners on the health status of the herd and allows the design of proactive pathogen control protocols, which is of particular interest for PRRSV. Surveillance of the virus on farms allows to determine the herd status regarding PRRSV shedding and exposure. There is a need for higher diagnostic sensitivity for increased reliability of the status. Two pillars of diagnostic sensitivity are the analytical sensitivity of the test and the number of pigs sampled in the epidemiological unit. RT-qrtPCR is one of the most commonly used tests for the diagnosis of PRRSV shedding because of its high sensitivity and specificity [15]. Sampling collective OF is a way to dramatically increase the number of pigs sampled, while collection is easy, quick and stress-free for pigs and humans. The use of OF for the diagnosis of PRRSV has been well documented for growing and adult pigs, but studies are lacking in weaning-age piglets even though this is an important subpopulation to determine the PRRSV herd status. Kittawornrat et al. [25] described the PRRSV-2 shedding in OF using litter-based collection with a cotton rope presented to weaning-age piglets. In our knowledge, our work is the first one exploring PRRSV-1 shedding in suckling piglets’ OF.

The objective of our field study was to compare the ability of detection of PRRSV-1 using three different samples. In our study, the true PRRSV status of litters was unknown because not all piglets were bled. Thus the sensitivity couldn’t be calculated for each index test because of the lack of an accepted gold standard. We only aimed to compare the interest of OF samples with the usual protocol based on blood sampling at weaning in one piglet per litter [8].

PRRSV RNA could be detected in all types of sample tested with RT-qrtPCR. Replication in local tissue such as salivary glands and tonsils may explain the presence of PRRSV in OF [18]. In our study, we observed a high correlation for the detection of PRRSV-positive litters between serum sampled from the weakest piglets within the litter and cOF. However, less PRRSV RNA was found in cOF compared to serum. The viral load analysis based on Ct suggests that the ability of detection of PRRSV in serum and cOF assays, although similar, are based on different mechanisms. RT-qrtPCR on serum samples had a lower Ct and therefore a probably higher viral load than cOF. The ability to detect PRRSV RNA is therefore higher for serum. However, not all piglets in a litter were necessarily viraemic, so a unique serum sample was not always sufficient to determine PRRSV status of a litter. On the other hand, using cOF involves a higher number of animals sampled, as most piglets chew the rope. This increase in the number of animals sampled increased the chance to detect PRRSV RNA in a litter. This hypothesis is reinforced by the fact that the viral load from cOF was statistically higher if the serum of the weakest piglet of the litter was positive while the opposite does not apply. This implied that the virus concentration in a cOF sample should be linked to the number of positive piglets in the litter. This result was consistent with De Regge [4] who demonstrated in post-weaning pigs that the probability to detect PRRSV in a pen-based OF sample is correlated with the percentage of RT-qrtPCR-positive pigs for serum per pen.

In our study, PRRSV detection in iOF collected from the weakest piglet of each litter had a significantly lower rate of detection of PRRSV compared to serum and cOF. According to our hypothesis described above, the viral load in iOF may be low, even under the detection threshold. These results are in accordance with previous studies, which found that iOF had a lower detection rate than serum samples which had the best performance during the acute phase of infection [15].

Lower viral loads in OF might also be explained by technical issues with OF matrix. For PRRSV-2, it has been demonstrated that untreated cotton is the best matrix for OF collection [28], and that samples are best kept refrigerated until analysis [13]. In our study, these recommendations were respected. The OF samples were cooled immediately after collection and kept in cool storage until analysis at the laboratory. Saliva is known to contain inhibitors that can reduce analytical sensitivity [15]. RNA is rapidly degraded in OF and analysis pre-processing can therefore be critical. Centrifugation will sediment large particles such as feed fragments, but is insufficient to remove enzymes or others proteins [29]. Centrifugation of OF at speeds of up to 15,000 g for 15 min resulted in increased sensitivity of PCR for the lowest viral loads and reduced the mean Ct of samples [29]. In our study, OF samples were centrifuged at 1000 g for 10 minutes and it can be debated whether this was sufficient to obtain a good quality RNA and perform PRRSV sequencing. Other publications have divergent opinions on the usefulness of centrifugation [25, 28, 30].

Finally, the effects of pooling serum and OF samples on PRRSV detection were evaluated. In order to test a large number of pigs, pooled samples analysis were routinely used for the monitoring of PRRS. Samples pooling could also result in major savings of consumables and time, thereby reducing the cost of analysis [15]. Nevertheless, pooling decreased detection level and should therefore be used judiciously. The pooling of positive and negative samples may reduce PRRSV detection level due to dilution of the RNA [31]. An estimated 6% of positive samples would be missed if pools of five serum samples were used to detect PRRSV [31]. Another study described a more significant decrease in sensitivity for PRRSV detection for pools of five samples [15]. In our study, when containing at least one positive individual sample, 67% of pooled sera and 58% of pooled cOF samples were detected positive. This difference might be explained by the study design of Gerber et al., where pigs were inoculated and sampled early after challenge while our study was conducted under field conditions. The virulence of PRRSV isolates may also influence the number of samples that could be pooled in the same RT-qrtPCR reaction [30]. In our study, the rate of detection after pooling was sufficient at batch level for detecting PRRSV.

The sequencing of PRRSV was conducted at the end of the study. The Ct of RT-qrtPCR on cOF was lower than that on serum and was insufficient to sequence the virus. The cOF should therefore not be the only sample taken if sequencing is required, particularly on MLV vaccinated farms. As pooling decreases the viral load of the samples, sequencing should preferably be performed on an individual positive serum sample.

## Conclusions

Our study is the first to aim detecting PRRSV-1 RNA on OF samples in weaning-age piglets from a farm infected by PRRSV using RT-qrtPCR. It shows that when assessing viral circulation in a litter of suckling piglets: (i) the rate of detection of PRRSV-1 in cOF samples was similar to that in serum; (ii) virus sequencing, if required, should be performed on individual serum samples; (iii) the smaller the Ct of a cOF sample from a litter, the greater the likelihood that the serum sample from a piglet of that litter is positive.

A cost-effective and representative sampling protocol to monitor sow herds stabilisation could be as follows:

1. collect both cOF and one serum sample per litter
2. first, perform PCR on pooled cOF
3. if results are negative, the batch can be considered negative
4. if results are positive in a unvaccinated herd or a killed vaccine vaccinated one, the batch is considered positive
5. if results are positive in a herd vaccinated with a MLV, serum samples of litters with positive cOF should be tested for sequencing (selecting the litters with the lowest Ct for cOF)

## Abbreviations

CI: Confidence Interval
cOF: Collective Oral Fluid
Ct: Threshold Cycle
iOF: Individual Oral Fluid
MLV: Modified Live Vaccine
OF: Oral Fluid
ORF: Open Reading Frame
PRRS: Porcine Reproductive and Respiratory Syndrome
PRRSV: Porcine Reproductive and Respiratory Syndrome Virus
RNA: Ribonucleic Acid
RT-qrtPCR: Reverse Transcriptase Quantitative Real Time Polymerase Chain Reaction

## References

[1] Zimmerman J, Karriker LA, Ramirez A, Schwartz K, Stevenson GW (2012). Porcine reproductive and respiratory syndrome virus (porcine arterivirus). Diseases of Swine.

[2] Neumann EJ, Kliebenstein JB, Johnson CD, Mabry JW, Bush EJ, Seitzinger AH (2005). Assessment of the economic impact of porcine reproductive and respiratory syndrome on swine production in the United States. J Am Vet Med Assoc.

[3] Nathues H, Alarcon P, Rushton J, Jolie R, Fiebig K, Jimenez M (2017). Cost of porcine reproductive and respiratory syndrome virus at individual farm level – an economic disease model. Prev Vet Med..

[4] De Regge N, Cay B (2016). Comparison of PRRSV nucleic acid and antibody detection in pen-based Oral fluid and individual serum samples in three different age categories of post-weaning pigs from endemically infected farms. PLoS One.

[5] Stadejek T, Stankevicius A, Murtaugh MP, Oleksiewicz MB (2013). Molecular evolution of PRRSV in Europe: current state of play. Vet Microbiol.

[6] Berton P, Normand V, Martineau G-P, Bouchet F, Lebret A, Waret-Szkuta A. Evaluation of porcine reproductive and respiratory syndrome stabilization protocols in 23 French Farrow-to-finish farms located in a high-density swine area. Porc Health Manag. 2017 [cited 13 Jul 2018];3(1). Available from: http://porcinehealthmanagement.biomedcentral.com/articles/10.1186/s40813-017-0058-1. 10.1186/s40813-017-0058-1 PMC5440989 28546868

[7] Gouvars B, Auvigne V, Stadejek T, Sellal E. PRRS strain diversity in a European pig production area 14 years after the primary infection. In: Proc 21th IPVS Congress. Vancouver, Canada; 2010. p. 500.

[8] Holtkamp DJ, Morrison B, Rowland RR, Snelson H (2011). Terminology for classifying swine herds by porcine reproductive and respiratory syndrome virus status. J Swine Health Prod.

[9] Cano JP, Dee SA, Murtaugh MP, Rovira A, Morrison RB (2009). Infection dynamics and clinical manifestations following experimental inoculation of gilts at 90 days of gestation with a low dose of porcine reproductive and respiratory syndrome virus. Can J Vet Res.

[10] Atkinson JC, Dawes D, Ericson T, Fox PC, Gandara BK, Malamud D (1993). Guidelines for saliva nomenclature and collection. Ann N Y Acad Sci.

[11] Dawson LL, Edwards SA (2015). The effects of flavored rope additives on commercial pen-based oral fluid yield in pigs. J Vet Behav Clin Appl Res.

[12] Rai B. Oral Fluid in Toxicology. Internet J Toxicol. 2006 [cited 10 Jul 2018];3(2). Available from: http://ispub.com/IJTO/3/2/8140.

[13] Prickett JR, Zimmerman JJ (2010). The development of oral fluid-based diagnostics and applications in veterinary medicine. Anim Health Res Rev.

[14] Rotolo ML, Sun Y, Wang C, Giménez-Lirola L, Baum DH, Gauger PC (2017). Sampling guidelines for oral fluid-based surveys of group-housed animals. Vet Microbiol.

[15] Gerber PF, O’Neill K, Owolodun O, Wang C, Harmon K, Zhang J (2013). Comparison of commercial real-time reverse transcription-PCR assays for reliable, early, and rapid detection of heterologous strains of porcine reproductive and respiratory syndrome virus in experimentally infected or noninfected boars by use of different sample types. J Clin Microbiol.

[16] Pepin B, Liu F, Main R, et al. Collection of oral fluid from individually housed sows. J Swine Health Prod. 2015;23(1):35–37.

[17] Pol F, Dorenlor V, Eono F, Eudier S, Eveno E, Liégard-Vanhecke D (2017). Individual and pen-based oral fluid sampling: a welfare-friendly sampling method for group-housed gestating sows. Prev Vet Med..

[18] Decorte I, Van Campe W, Mostin L, Cay AB, De Regge N (2015). Diagnosis of the Lelystad strain of porcine reproductive and respiratory syndrome virus infection in individually housed pigs: comparison between serum and oral fluid samples for viral nucleic acid and antibody detection. J Vet Diagn Investig.

[19] Kittawornrat A, Zimmerman JJ (2011). Toward a better understanding of pig behavior and pig welfare. Anim Health Res Rev.

[20] Docking CM, de Weerd HAV, Day JEL, Edwards SA (2008). The influence of age on the use of potential enrichment objects and synchronisation of behaviour of pigs. Appl Anim Behav Sci.

[21] Kittawornrat A, Prickett J, Wang C, Olsen C, Irwin C, Panyasing Y (2012). Detection of porcine reproductive and respiratory syndrome virus (PRRSV) antibodies in oral fluid specimens using a commercial PRRSV serum antibody enzyme-linked immunosorbent assay. J Vet Diagn Investig Off Publ Am Assoc Vet Lab Diagn Inc.

[22] Seddon YM, Guy JH, Edwards SA (2012). Optimising oral fluid collection from groups of pigs: effect of housing system and provision of ropes. Vet J Lond Engl 1997.

[23] Fablet C, Renson P, Pol F, Dorenlor V, Mahé S, Eono F (2017). Oral fluid versus blood sampling in group-housed sows and finishing pigs: feasibility and performance of antibody detection for porcine reproductive and respiratory syndrome virus (PRRSV). Vet Microbiol.

[24] Lopez WA (2018). Porcine reproductive and respiratory syndrome monitoring in breeding herds using processing fluids. J Swine Health Prod..

[25] Kittawornrat A, Panyasing Y, Goodell C, Wang C, Gauger P, Harmon K (2014). Porcine reproductive and respiratory syndrome virus (PRRSV) surveillance using pre-weaning oral fluid samples detects circulation of wild-type PRRSV. Vet Microbiol.

[26] Mateu E, Martin M, Vidal D (2003). Genetic diversity and phylogenetic analysis of glycoprotein 5 of European-type porcine reproductive and respiratory virus strains in Spain. J Gen Virol.

[27] Oleksiewicz MB, Bøtner A, Madsen KG, Storgaard T (1998). Sensitive detection and typing of porcine reproductive and respiratory syndrome virus by RT-PCR amplification of whole viral genes. Vet Microbiol.

[28] Olsen C, Karriker L, Wang C, Binjawadagi B, Renukaradhya G, Kittawornrat A (2013). Effect of collection material and sample processing on pig oral fluid testing results. Vet J Lond Engl 1997.

[29] Gibert E, Martín-Valls G, Mateu E (2017). Comparison of protocols for the analysis of type 1 porcine reproductive and respiratory syndrome virus by RT-PCR using oral fluids. J Virol Methods.

[30] Ramirez A, Wang C, Prickett JR, Pogranichniy R, Yoon K-J, Main R (2012). Efficient surveillance of pig populations using oral fluids. Prev Vet Med.

[31] Rovira A, Clement T, Christopher-Hennings J, Thompson B, Engle M, Reicks D (2007). Evaluation of the sensitivity of reverse-transcription polymerase chain reaction to detect porcine reproductive and respiratory syndrome virus on individual and pooled samples from boars. J Vet Diagn Investig.

